# Supplementary material for: The causal relationship between the human gut microbiota and pyogenic arthritis: a Mendelian randomization study
Source: Front Cell Infect Microbiol. 2024 Nov 26;14:1452480. doi: 10.3389/fcimb.2024.1452480 (PMC11629706; doi:10.3389/fcimb.2024.1452480)
Supplement: Supplementary file 1 [file DataSheet1.zip › mendelian test/ebi-a-GCST90027599.csv_forest.pdf]

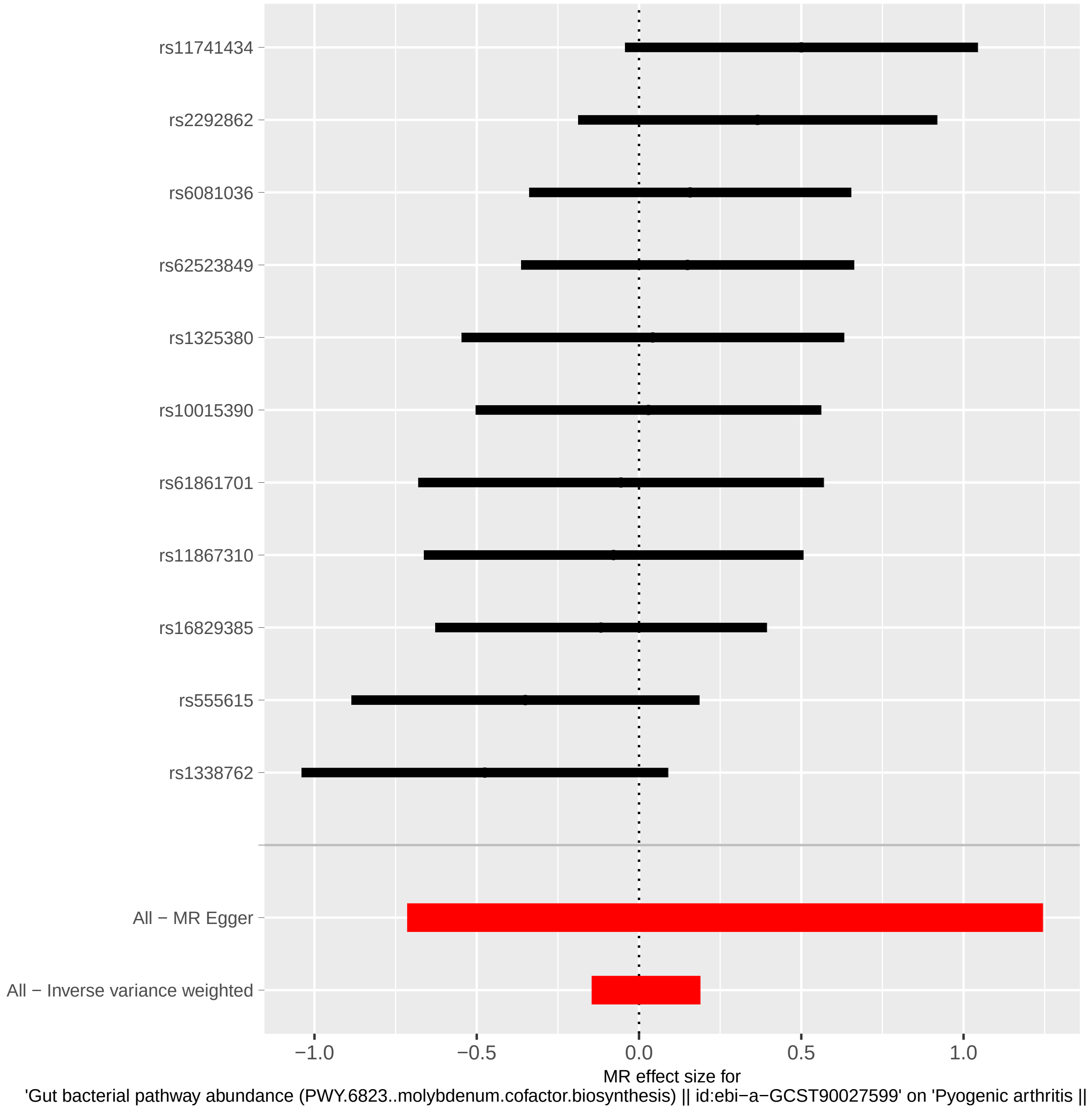

rs11741434
rs2292862
rs6081036
rs62523849
rs1325380
rs10015390
rs61861701
rs11867310
rs16829385
rs555615
rs1338762
All − MR Egger
All − Inverse variance weighted
−1.0
−0.5
0.0
0.5
1.0
MR effect size for
'Gut bacterial pathway abundance (PWY.6823..molybdenum.cofactor.biosynthesis) || id:ebi−a−GCST90027599' on 'Pyogenic arthritis ||
